# Proteomics Analysis to Identify Proteins and Pathways Associated with the Novel Lesion Mimic Mutant E40 in Rice Using iTRAQ-Based Strategy

**DOI:** 10.3390/ijms20061294

**Published:** 2019-03-14

**Authors:** Xiang-Bo Yang, Wei-Long Meng, Meng-Jie Zhao, An-Xing Zhang, Wei Liu, Zhao-Shi Xu, Yun-Peng Wang, Jian Ma

**Affiliations:** 1 Faculty of Agronomy, Jilin Agricultural University, Changchun 130118, China; yangxiangbo1980@163.com (X.-B.Y.); mengweilongosj@163.com (W.-L.M.); zhanganxingosj@163.com (A.-X.Z.); liuweiosj@163.com (W.L.); 2 Institute of Agricultural Biotechnology, Jilin Academy of Agricultural Sciences, Changchun 130033, China; 3 Institute of Crop Sciences, Chinese Academy of Agricultural Sciences (CAAS)/National Key Facility for Crop Gene Resources and Genetic Improvement, Key Laboratory of Biology and Genetic Improvement of Triticeae Crops, Ministry of Agriculture, Beijing 100081, China; zhao_mengjie0815@163.com (M.-J.Z.); xuzhaoshi@caas.cn (Z.-S.X.)

**Keywords:** lesion mimic mutant, leaf spot, phenylpropanoid biosynthesis, proteomics, isobaric tags for relative and absolute quantitation (iTRAQ), rice

## Abstract

A novel rice lesion mimic mutant (LMM) was isolated from the mutant population of Japonica rice cultivar Hitomebore generated by ethyl methane sulfonate (EMS) treatment. Compared with the wild-type (WT), the mutant, tentatively designated E40, developed necrotic lesions over the whole growth period along with detectable changes in several important agronomic traits including lower height, fewer tillers, lower yield, and premature death. To understand the molecular mechanism of mutation-induced phenotypic differences in E40, a proteomics-based approach was used to identify differentially accumulated proteins between E40 and WT. Proteomic data from isobaric tags for relative and absolute quantitation (iTRAQ) showed that 233 proteins were significantly up- or down-regulated in E40 compared with WT. These proteins are involved in diverse biological processes, but phenylpropanoid biosynthesis was the only up-regulated pathway. Differential expression of the genes encoding some candidate proteins with significant up- or down-regulation in E40 were further verified by qPCR. Consistent with the proteomic results, substance and energy flow in E40 shifted from basic metabolism to secondary metabolism, mainly phenylpropanoid biosynthesis, which is likely involved in the formation of leaf spots.

## 1. Introduction

Some plant mutants spontaneously exhibit characteristics of typical pathogen infection without any pathogen attack, these mutants are termed lesion mimic mutants (LMMs). Most LMM gene mutations involve regulatory genes of immune responses or promoters of such genes, thereby constitutively expressing pathogenicity-related (PR) genes, causing the production of reactive oxygen species (ROS) and the accumulation of phytoalexins. LMMs are subjected to the development of spontaneous cell death and necrotic lesions. Therefore, LMMs are useful as genetic tools to investigate the molecular mechanisms of programmed cell death (PCD) and hypersensitive response (HR) in plants. Since Sekiguchi discovered the first LMM of rice in 1965 [1], numerous LMMs and the involved genes have been identified in many plant species, such as *Arabidopsis*, maize, groundnut, barley, and wheat [2,3,4,5,6,7,8,9]. 

Rice is a major staple food crop for a large part of the world’s population and an important model monocot plant species for research. Rice has a small genome size and the complete genome sequence is available [10,11]. LMMs in rice have been widely studied and more than 200 LMMs have been reported in rice so far. Most LMM mutation sites occur in the regions of regulatory genes that are resistant to pathogen infection [9,12,13,14,15,16]. These mutant genes are usually recessive genes and only a fraction are dominant or semi-dominant genes [17]. So far, at least 56 genes responsible for rice LMMs have been identified and registered in the Gramene database (http://www.gramene.org, accessed on: 11 August 2018). These LMM genes encode various functional proteins (Table 1). 

However, studies on LMMs often focus on mining disease resistance genes, relatively few has been reported on the plant immune response process and the optimum growth and development of plants in terms of the distribution of energy and matter. LMMs generally show a series of defective phenotypes such as reduced photosynthesis, decreased biomass accumulation, and severe yield reduction. These phenotypes are not caused by pathogen attack but are due to the abnormal activation of the immune system in LMMs, leading to redistribution of energy and substances in the metabolic pathways in the plants. In order to reveal changes in energy flow of the plant’s immune system, we need a more comprehensive proteomics study of LMMs mutants. Proteomics based on two-dimensional gel electrophoresis (2-DE) is one of the most commonly used strategies to identify differentially accumulated proteins between the wild-type (WT) rice and its LMMs. For example, two PR proteins, OsPR5 and OsPR10, and three ROS-scavenging enzymes, catalase (CAT), ascorbate peroxidase (APX), and superoxide dismutase (SOD), were differentially expressed in the blm mutant [30]. Similarly, peroxidase, thaumatin-like protein and probenazole-induced protein (PBZ1) were upregulated in the spl1 mutant [31]. However, the technical limitations of 2-DE make it difficult to identify the LMM-involving proteins at the whole proteome level. Only a limited number of differential proteins have been detected between LMMs and WT by 2-DE (e.g., about 18 by Kim et al. [31], 37 by Tsunezuka et al. [32], 33 by Jung et al. [30], and 159 by Kang et al. [33]) which greatly limits the development of proteome research on LMMs. Given its high resolution and accurate detailed protein expression profiles, iTRAQ combined with LC-MS/MS is widely used, and the accumulation of bioinformatics knowledge has made it possible to rapidly analyze and display data more accurately [34]. iTRAQ-based proteomics has been widely applied in investigating abiotic and biotic stresses response in plants [35,36], but is rarely reported in studies on mechanisms of LMMs. 

In this study, a novel rice lesion mimic mutant, E40, was isolated from the EMS mutant population of japonica rice cultivar Hitomebore. We performed proteomic analysis with the iTRAQ method using leaves from E40 plants at the four or five leaf stage to reveal the molecular mechanisms of lesion mimic phenotypes. A total of 2722 proteins were identified, among which 233 proteins were found to be differentially expressed. These proteins were analyzed to increase our understanding of the altered pathways between E40 and WT and the mechanisms involved in formation of lesions. 

## 2. Results

### 2.1. Phenotypic Characterization of E40

**Figure 1:**
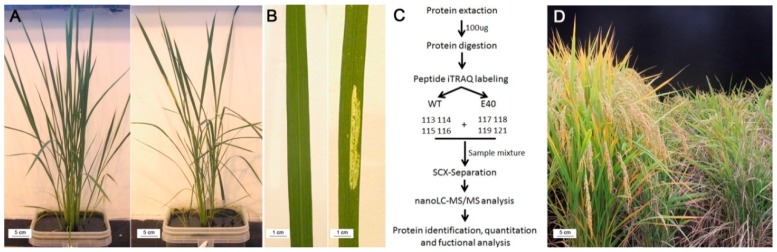
Phenotypic characterization of E40 and the experimental scheme of isobaric tags for relative and absolute quantitation (iTRAQ) analysis. (**A**) the phenotypes of paddy-grown wild type (WT, left) and E40 (right) plants grown in the greenhouse at tillering stage; (**B**) the phenotypes of WT (left) and E40 (right) leaves collected from plants in (**A**) showing the lesion mimic phenotypes of E40; (**C**) experimental scheme of sampling and iTRAQ analysis; (**D**) the phenotypes of WT (left) and E40 (right) grown in the field at the maturity stage.

In the greenhouse, E40 plants began to show visible lesion mimic spots in the middle parts of blades at the four- or five-leaf stage with increasing size and density during the later stages of plant development and at tillering stage. Meanwhile, the phenotypes in plant growth and development were significantly different between E40 and WT (Figure 1A,B). Therefore, we hypothesized that the protein difference between E40 and wild-type rice could reach the maximum divergence point during this period, and then the leaves of that stage were taken as samples of the iTRAQ experiment. In the field, the agronomic traits of E40 plants were even worse than indoors. All leaves of the E40 plants were wilted at the late flowering stage, as if they had been burned by sunlight, and plants eventually exhibited early senescence. Compared with the WT plants, the E40 plants displayed abnormal developmental phenotypes and lower agronomic trait values, including significantly reduced dry weight, plant height, number of panicles, grain number, and 1,000-grain weight were significantly reduced compared with WT (Table 2). From the tillering stage, E40 and WT began to show significant differences in dry weight. Until the fruiting period, the difference reached the maximum, nearly three times the difference in the field, and nearly twice the difference in the greenhouse.

### 2.2. Proteomics Analysis of Differentially Abundant Proteins between E40 and WT

The WT and E40 leaves at the tillering stage were harvested for iTRAQ analysis following the procedure shown in Figure 1C and 2722 proteins were identified from 25,887 distinct detected peptides (Additional File 1). Under the screening criteria of fold change greater than 1.5 or less than 0.67 and *p* value <0.05, a total of 233 proteins were identified to be differentially abundant expressed by comparison between E40 and WT; these proteins were regarded as candidate proteins associated with lesion formation (Table 3 and Supplementary Material: Table S1). Among them, 109 proteins were up-regulated and 124 were repressed. 

### 2.3. Gene Ontology (GO) Analysis of Altered Proteins

**Figure 2:**
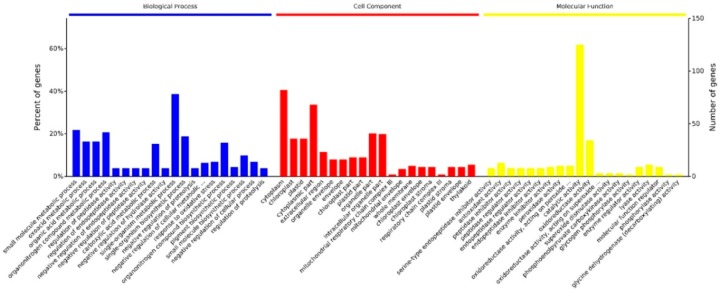
Gene Ontology (GO) distribution analysis. GO analysis could be clustered into three terms: the blue column chart represents biological processes term, the red column represents cellular component term, and the yellow column represents molecular function term. The number of proteins for each GO annotation is shown on the right axis, and the percent of proteins for each GO annotation is on the left axis. P values were calculated using a modified Fisher’s exact test and corrected for multiple testing using the Bonferroni correction in Omicsbean.

All the differentially abundant proteins identified were analyzed for gene ontology (GO) using GO Slim and classified as biological processes, cellular component, or molecular function (Figure 2). In the cell component group, the differentially abundant proteins were mainly distributed in the cytoplasm, chloroplast, and plastid. The molecular functional analysis indicated that catalytic proteins and those related to oxidoreductase activity were predominant. In terms of biological processes, identified proteins were mainly involved in metabolic process, regulation of enzyme activity, and response to oxidative stress. 

### 2.4. Kyoto Encyclopedia of Genes and Genomes (KEGG) Analysis

**Figure 3:**
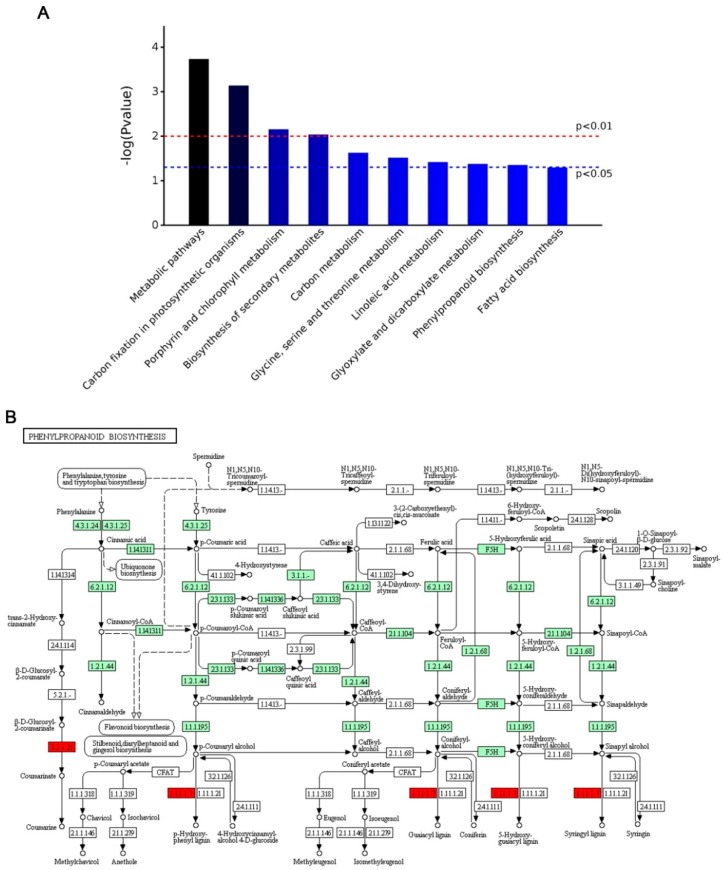
Enriched Kyoto Encyclopedia of Genes and Genomes (KEGG) pathway-based sets and a diagram of phenylpropanoid biosynthesis. (**A**) KEGG pathways which the differentially abundant proteins were enriched. The x-axis shows the proteins involved in the extended KEGG network and pathway. P values were calculated using a modified Fisher’s exact test and corrected for multiple tests using the Bonferroni correction in Omicsbean. (**B**) A diagram of phenylpropanoid biosynthesis. Enzymes in red indicate that the corresponding proteins were up-regulated, and those painted green indicate that the proteins were not significantly up- or down-regulated in E40 compared with WT. Up-regulated protein: 1.11.1.7, Peroxidase; 3.2.1.21, Beta-glucosidase.

Pathway analysis using the Kyoto Encyclopedia of Genes and Genomes (KEGG) pathway database (http://www.genome.jp/kegg/pathway.html, accessed on: 08 November 2018) identified 9 pathways (*p* < 0.05) related to these proteins with differential relative abundance, as shown in Figure 3A. Most pathways related to basic metabolism were down-regulated, including carbon fixation, linoleic acid metabolism, carbon metabolism, and amino acid biosynthesis. The only uniquely up-regulated pathway was phenylpropanoid biosynthesis, which is related to secondary metabolism (Figure 3B). 

### 2.5. Protein-Protein Interaction Analysis

**Figure 4:**
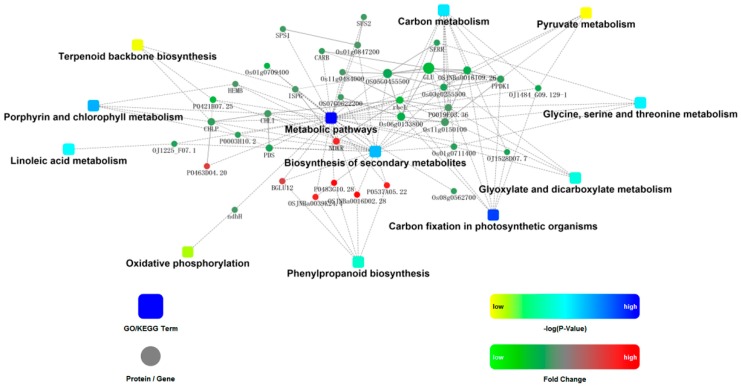
Protein-protein interaction (PPI) network among differentially abundant proteins. The following network model was generated with Cytoscape based on information gained from up to three levels of functional analysis: fold change of gene/protein, protein–protein interaction, and KEGG pathway enrichment. Circle nodes denote genes/proteins, and rectangles denote the KEGG pathway or biological process. P values, mean Pathways related to proteins with differential relative abundance, are colored with gradient colors from yellow to blue from 7.66 × 10^−2^ to 1.85 × 10^−4^. Yellow denotes a low P value and blue denotes a high P value.

In order to get more information and discover the mechanisms involved in formation of lesion phenotypes, the involved proteins in nine pathways with P values <0.05 and three additional pathways including terpenoid backbone biosynthesis, pyruvate metabolism, and oxidative phosphorylation were analyzed using STRING for protein-protein interaction (PPI) analysis (Figure 4). 

The central network showed that proteins such as PPDK1 (Q6AVA8), Glu (Q69RJ0), rbcL (P0C512), CHL (Q53RM0), and OSJNBa0016I09.26 (Q5QNA5) were nodes; they interacted with each other and other proteins related to most pathways including porphyrin and chlorophyll metabolism, carbon fixation in photosynthetic organisms, linoleic acid metabolism, glyoxylate and dicarboxylate metabolism, and terpenoid backbone biosynthesis. Proteins related to phenylpropanoid biosynthesis and oxidative phosphorylation interacted indirectly with the above pathways through basal metabolism and secondary metabolism. 

### 2.6. qRT-PCR Data are Consistent with Protein Abundance Analysis Revealed by iTRAQ

To complement the iTRAQ results, eight genes were chosen for expression assays by real-time PCR (RT-PCR). As shown in Table 4, four genes encoding Q5JMS4, Q7XSV2, Q94DM2, and Q9AS12 involved in phenylpropanoid biosynthesis were all up-regulated, which are consistent with iTRAQ results, showing that the four proteins were more abundant in E40 plants. Besides, the results of qRT-PCR and protein abundance assays were consistent on the two genes involved in porphyrin and chlorophyll metabolism, and two genes involved in carbon fixation in photosynthetic organisms. The consistency between RT-PCR and iTRAQ results indicates the reliability of these data (Table 4). 

## 3. Discussion

Like other LMMs, LMM rice E40 was obtained by EMS treatment and displayed lower height, fewer tillers, lower yield, and premature death compared to WT. A total of 233 differentially abundant proteins were identified containing 109 up-regulated proteins and 124 down-regulated proteins. KEGG analysis showed all these 233 proteins were enriched in 9 signal pathways (*p* < 0.05) including metabolic pathways, carbon fixation in photosynthetic organisms, porphyrin and chlorophyll metabolism, biosynthesis of secondary metabolites, carbon metabolism, glycine, serine and threonine metabolism, linoleic acid metabolism, glyoxylate and dicarboxylate metabolism, and phenylpropanoid biosynthesis. Almost all the pathways were down-regulated except for phenylpropanoid pathway. 

Glycine, serine and threonine are indispensable amino acid in plants and participate in multiple synthesis of important substances, and the change of glycine, serine and threonine metabolism could affect the basic substance synthesis and metabolic processes. Numerous serine-threonine protein kinases universally excited in plants and could regulate signaling transduction pathways via phosphorylated threonine and serine of target proteins [37,38]. Glycine could promote the absorption of phosphorus, improve plant stress resistance, and promote plant growth, especially photosynthesis. Serine is related to lignin biosynthesis, PCD, and plant aging [39]. And transport factors also have impact on expression of relative genes involving in transduction pathways [40]. Abnormal metabolism could lead to disturbance of signaling pathway. In our study, it is worth noting that the expression levels of chitinase and some enzymes related to lignin biosynthesis were enhanced in E40. Mauch et al. [41] found that chitinase function in defense against fungus, which could degrade fungal cell walls. Lignin is accumulated and deposited in the cell wall, and this accumulation could enhance the ability of the cell wall to resist the invasion of pathogenic microorganisms and provide mechanized protection for the plasma membrane-wrapped protoplasm [42]. Lignin biosynthesis can thus be triggered as a response to various biotic and abiotic stresses in cells. Evidence has shown that lignin biosynthesis genes play crucial roles in basal defense and normal growth of plants [43]. In addition, salt stress induced protein was also up-regulated (Table 3). Thus, we inferred that the up-regulation of chitinase, enzymes related to lignin biosynthesis, and salt stress induced protein were caused by the immune disorder in E40.

EMS treatment could cause the mutation of specific plant immune regulatory genes. This resulted in abnormal expression of plant immune response-related genes, causing an increase in ROS in vivo, accompanied by intracellular peroxides. Plant secondary metabolic pathways significantly increased in E40. The only up-regulated pathway in E40 was the phenylpropanoid pathway (Figure 4). Phenylpropanoids are involved in biotic stress response in plants and the phenylpropanoid pathway plays a critical role in the plant innate immune system, which produces a variety of secondary metabolites, such as flavonoids, isoflavonoids, lignin, anthocyanin, phytoalexins, and phenolic esters; all of these are critical in development, structural protection, defense responses, and tolerance to abiotic stimuli [44,45]. Previous studies showed that enzymes in the phenylpropanoid biosynthetic pathway are associated with PCD [30,46]. Thus, it most likely leads to formation of PCD and the lesion mimic phenotype. 

The main carbon source for the phenylpropanoid pathway is phenylalanine, which is synthesized by erythrose-4-phosphate (E4P) from the pentose phosphate pathway and phosphoenolpyruvate (PEP) from the glycolysis pathway. E4P and PEP are also important intermediates for maintaining normal operation of basic metabolism through a stable supply of both material and energy. A strengthened phenylpropanoid pathway means a strong demand for E4P and PEP, which will affect normal metabolism in E40. More PEP from glycolysis leads to less pyruvate and means fewer carbon skeletons are generated for amino acid biosynthesis and energy release from the following tricarboxylic acid cycle (TCA) and that there are fewer substrates for carbon dioxide fixation in photosynthesis (in C4 plants). Demand for more E4P from the pentose phosphate pathway leads to greater demands of pentose, meaning less ribose-5-phosphate for nucleotide formation and resulting in deficient biosynthesis of adenosine triphosphate (ATP), coenzyme A (CoA), nicotinamide adenine dinucleotide (NAD), Flavine adenine dinucleotide (FAD), RAN, and DNA, and fewer ribulose-5-phosphate for carbon fixation in photosynthesis as well. 

An important and complex consequence of an abnormal increase in the phenylpropanoid pathway lies in the effects on porphyrin and chlorophyll metabolism. On the one hand, as the biosynthesis of chlorophyll begins from glutamic acid transformed from α-ketoglutarate, an intermediate product of TCA, the increased phenylpropanoid pathway results in imbalance of the TCA cycle, leading to an energy supply shortage and insufficient substrate for porphyrin and chlorophyll synthesis. Tight regulation of chlorophyll biosynthesis and degradation is essential to cell survival [47]. Disruption of chlorophyll biosynthesis at different stages can lead to lesion-mimic phenotypes due to the abnormal accumulation of photoreactive molecules, like porphobilinogen [48], coproporphyrinogen III [28], uroporphyrinogen III [49], and protochlorophyllide [50]. On the other hand, the insufficient chlorophyll supply leads to low energy supply (from photosystem I and II) for synthesizing photosynthetic proteins. Combined with the shortage of PEP for carbon fixation, the turnover of damaged photosynthetic proteins in photoinhibition is suppressed; this results in ROS accumulation. Accumulation of ROS causes the loss of chloroplast integrity followed by rupture of the central vacuole and, finally, cell death [51]. Therefore, ROS regulators, e.g., peroxidases, oxidoreductases, and antioxidant proteins, are increased in E40, a typical feature of LMMs. 

**Figure 5:**
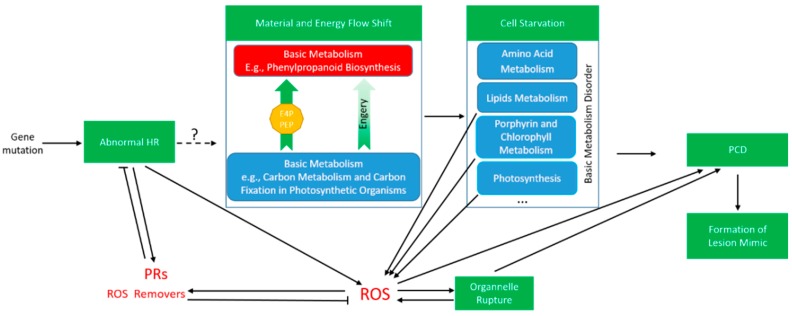
Putative mechanism of formation of lesion mimic in E40. HR, hypersensitive response; PCD, programmed cell death; PRs, pathogen-related proteins; ROS, reactive oxygen species.

Carbon fixation is an important biological process in plant photosynthesis [52]. Porphyrin is an intermediate substance of biosynthesis of chlorophyll [53], both of which are closely related to the photosynthesis efficiency and directly affect plant biomass. E40 displayed lower height, fewer tillers, lower yield is most likely to result from the down-regulated carbon fixation pathway and porphyrin and chlorophyll metabolism. Moreover, PPI analysis showed that terpenoid backbone biosynthesis are linked to porphyrin and chlorophyll metabolism, carbon fixation in photosynthetic organisms, linoleic acid metabolism, glyoxylate and dicarboxylate metabolism through the regulation of expression of node proteins (commonly protease). Many important growth regulators, such as ABA, carotenoids, and vitamin A, belonged to terpenoid, which play essential roles in seed generation, plant growth and development. Up-regulation of the phenylpropanoid biosynthesis pathway was accompanied by down-regulation of other pathways. We therefore concluded that the reason for lesion mimic in E40 is due to substance and energy flow shifting from basic metabolism to secondary metabolism, especially, phenylpropanoid (lignin) biosynthesis. As shown in Figure 5, a mutation caused abnormal HR that triggers: (1) a shift in substance and energy flow from basic metabolism to secondary metabolism, followed by cell starvation with suppression/interruption of basic metabolism such as porphyrin and chlorophyll synthesis, disordered metabolism directly or indirectly (via ROS accumulation) results in PCD, and, finally, formation of lesion mimic; (2) accumulation of ROS (also accumulated from disordered metabolism caused by cell starvation), the ROS then directly and/or indirectly (via organelle rupture) lead to PCD and finally formation of lesion mimic. 

## 4. Materials and Methods 

### 4.1. Plant Materials

The novel rice lesion mimic mutant E40 was identified among 4500 mutant lines generated by EMS treatment of the northern Japan japonica rice cultivar Hitomebore. E40 has been self-bred for five generations and stably displays the target trait in greenhouse and field conditions. Agronomic traits of WT and E40 were determined while M5 generation mutant and WT were grown in a paddy field in Changchun, Jilin Province, China in 2016. Seeds of M5 generation mutant and WT were planted in a greenhouse at 28/24 °C (day/night). At the tillering stage when the lesions were large enough, pictures were taken, and leaves were harvested for proteomic analysis. 

### 4.2. Protein Extraction

Approximately 100 mg of leaves from E40 and WT were thoroughly ground to a fine powder in liquid nitrogen, added with 1:10 (*w*/*v*) Lysis buffer (pH 8.5), containing 2 M thiourea, 7 M urea and 4% CHAPS with protease inhibitors (Sigma, St. Louis, MO, USA), for protein extraction at room temperature. The mixture was sonicated for 60 s to obtain more soluble proteins. The plant residue was removed by low temperature centrifugation, and the supernatant was transferred to a 50 cm^3^ tube containing 4 volumes of 10% (*w*/*v*) trichloroacetic acid (TCA)/acetone, mixed and stored at −20 °C overnight. The precipitated protein was collected by centrifugation at 40,000× *g* for 10 min at 4 °C and washed three times with cold acetone, and finally lyophilized. After removal of acetone protein was resuspended in lysis buffer. Protein concentration was determined by was detected by Nano Photometer spectrophotometer (IMPLEN, Westlake Village, CA, USA) Agilent Bioanalyzer 2100 system (Agilent Technologies, Santa Clara, CA, USA). The protein samples were stored at −80 °C.

### 4.3. Trypsin Digestion and iTRAQ Labeling

Each sample from different sources was treated in the manner described in the iTRAQ protocol (AB SCIEX, Redwood City, CA, USA), approximately 100 μg total protein was taken, centrifuged at 100,000× *g* for 15 min at 4 °C, dried and resuspended in 50 mm^3^ of lysis buffer. After reduction and cysteine-blocking, the proteins were digested with sequencing grade trypsin (50 ng/mm^3^) for 12 h at 37 °C, and finally 150 mm^3^ of an ethanol solution in which the iTRAQ reaction solution was dissolved was added to the reaction solution for labeling.

In the present study, an experiment setting of 4:4 (eight-plex) was selected. The four biological replicates of leaves from WT were labeled with 113, 114, 115, and 116 tags, and the four biological replicates of leaves from E40 were labeled with 117, 118, 119, and 121 tags (Figure 1C). After incubation at room temperature for 2 h and termination of the labeling reaction, the labeled samples were then mixed and dried with a rotary vacuum concentrator. 

### 4.4. LC-MS/MS and Bioinformatics Analysis

The labeled samples were separated at 0.3 cm^3^/min with a nonlinear binary gradient, and segments were prepared for LC-MS/MS analysis. Rare data acquisition was performed with a Triple TOF 5600 System (AB SCIEX, Redwood City, CA, USA) fitted with a Nanospray III source (AB SCIEX, Redwood City, CA, USA) and a pulled quartz tip as the emitter (New Objectives, Woburn, MA, USA), and protein identification and quantification were performed with Protein Pilot Software v. 5.0 (AB SCIEX, Redwood City, CA, USA) against the *Oryza sativa* database (https://www.ncbi.nlm.nih.gov/protein/?term=Oryza%20sativa, accessed on: 15 October 2018) using the Paragon algorithm. Detailed methods and parameter settings for these experiments followed the protocol detailed on the website https://www.ebi.ac.uk/pride/archive/projects/PXD005731, (accessed on: 15 October 2018). 

In order to narrow down the protein number and focus on the most significant proteins, the screening criteria of differential proteins were: fold change greater than 1.5 or less than 0.67 and *p* value <0.05. The mean value of several repetitions was calculated using two group samples relative quantitative value. Bioinformatics analysis was conducted using Quick GO (http://www.ebi.ac.uk/ QuickGO/, EMBL-EBI, Wellcome Genome Campus, Hinxton, Cambridgeshire, UK, accessed on: 22 November 2018), Ingenuity Pathway Analysis software (http://www.polyomics.gla.ac.uk/resource-ipa.html, Glasgow Polyomics, College of Medical, Veterinary and Life Sciences, accessed on: 30 November 2018), UniProt (http://www.uniprot.org/, Centre Medical Universitaire 1, rue Michel Servet, 1211 Geneva 4 Switzerland, accessed on: 13 March 2019), STRING (http://string-db.org/, ELIXIR, Wellcome Genome Campus, Hinxton, Cambridgeshire, UK, accessed on: 24 February 2019) and OmicsBean (http://www.omicsbean.cn/, Gene for health, Shanghai, China, accessed on: 18 December 2018). 

### 4.5. qRT-PCR Analysis

Total cellular RNA was extracted using TransZol Plant Kit (Transgen Biotech, Beijing, China) and treated with TransScript All-in-One First-Strand cDNA Synthesis SuperMix for qPCR Kit (Transgen Biotech, Beijing, China) for cDNA production. qRT-PCR was carried out using SYBR Green master mix (Transgen Biotech, Beijing, China) and specific primer sets (Table 4). Amplification reactions were performed under the following conditions: 2 min at 50 °C, 10 min at 95 °C, 40 cycles for 15 s at 95 °C, 1 min at 60 °C. Relative transcript levels were calculated using the 2^−ΔΔCT^ method as specified by the manufacturer. The relative expression values of the targeted gene were normalized to the expression value of the glyceraldehyde-3-phosphate dehydrogenase (*GAPDH*) gene. 

## 5. Conclusions

In summary, we carried out proteomics analysis to understand cell death and identify proteins activated in LMM mutant E40. A total of 233 proteins, screened from 2722 proteins identified using iTRAQ, exhibited differential abundance. The data was complemented by qRT-PCR analysis with randomly selected genes that encode differentially abundant proteins. The number of proteins identified in this study is larger than other reports about LMMs using the 2-DE method. Identified proteins are involved in diverse biological processes. Consistent with the proteomics results, we speculated that substance and energy flow shifted in E40 from basic metabolism to secondary metabolism, mainly phenylpropanoid biosynthesis, which is the main reason for formation of leaf spots. 

## Figures and Tables

**Table 1:** Some proteins encoded by lesion mimic mutant (LMM) genes.

Order	Functional Proteins	References
1	Heat stress transcription factor	[18]
2	U-Box/Armadillo repeat protein	[19]
3	Membrane-associated protein	[3,20]
4	Ion channel	[21]
5	Zinc finger protein	[21]
6	Acyltransferase	[22]
7	Ser/Thr protein kinase	[23]
8	Clathrin associated adaptor protein complex 1 medium subunit 1 (AP1M1)	[24]
9	Putative splicing factor 3b subunit 3 (SF3b3)	[25]
10	Proteins involved in biosynthesis pathways of fatty acids lipids	[26]
11	Aromatic amino acid	[27]
12	Porphyrin	[28]
13	Polyphenol oxidase enzyme in secondary metabolism	[29]

**Table 2:** Performance of agronomic traits of E40 cultured in the field and greenhouse.

	Material	Dry Weight (g)			Plant Height (cm)	No. of Panicle	Grain Number	1000-Grain Weight (g)
		Seedling Stage	Tillering Stage	Maturity Stage				
Field	E40	-	-	83.17 ± 7.71 **	98.7 ± 2.42 **	19.4 ± 4.62 *	32.25 ± 1.71 **	17.68 ± 0.45 **
	WT	-	-	247.62 ± 5.42	102.58 ± 1.05	23.2 ± 2.77	134.62 ± 2.84	23.6 ± 0.16
Greenhouse	E40	0.0224 ± 0.0017	4.15 ± 0.13 *	119.36 ± 4.76 **	115.23 ± 1.79 **	21.3 ± 1.72 *	47.05 ± 0.97 **	18.76 ± 0.42 **
	WT	0.0235 ± 0.0021	5.00 ± 0.06	218.67 ± 5.49	117.58 ± 2.16	23.9 ± 1.05	118.13 ± 2.47	22.96 ± 0.24

The data represent the means ± SD. Ten plants of each accession were evaluated for each agronomic trait. *, significance at *p* < 0.05, **, significance at *p* < 0.01.

**Table 3:** Twenty differentially abundant proteins associated with lesion formation in E40 compared with WT.

Protein ID	Gene Name	Annotation	Log2 Fold Change
Q8S059	SSI2	Stearoyl-[acyl-carrier-protein] 9-desaturase 2	−3.259320177
Q84ZD2	P0534A03.109	Pentatricopeptide repeat-containing protein CRP1 homolog	−2.235369295
Q2QVA7	LOC_Os12g13460	protein-lysine N-methyltransferase activity	−1.957389474
B9F2U5	Os02g0157700	Promotes chloroplast protein synthesis	−1.670452917
P0C512	rbcL	Ribulose bisphosphate carboxylase large chain	−1.525042871
Q69RJ0	GLU	Ferredoxin-dependent glutamate synthase	−1.459112364
O04882	P0421H07.25	Farnesyl diphosphate synthase	−1.418836635
Q5NAI9	P0456F08.15	Putative OsFVE	−1.411837321
Q69X42	P0429G06.10	glycine dehydrogenase (decarboxylating) activity	−1.372611128
Q0JJY1	Os01g0709400	hydrolase activity	−1.329396063
Q9LGB2	P0504H10.32	Putative wound-induced protease inhibitor	1.251971273
Q2QLS7	LOC_Os12g43450	P21 protein, putative	1.325084219
Q7XSU8	OSJNBa0039K24.8	Belongs to the peroxidase family	1.339045312
Q8W084	OSJNBa0091E23.10	Putative pathogenesis-related protein	1.397933437
Q0JR25	RBBI3.3	Bowman-Birk type bran trypsin inhibitor	1.417877593
Q9AWV5	P0044F08.5	serine-type endopeptidase inhibitor activity	1.447168008
Q5WMX0	dip3	Putative chitinase	1.502285455
Q0JMY8	SALT	Salt stress-induced protein	1.596525805
Q75GR1	OSJNBb0065L20.2	*	1.728124034
Q306J3	JAC1	Dirigent protein	1.770280249

Note: *, uncharacteristic protein.

**Table 4:** Gene-specific primers used for the real-time PCR.

Pathway	Protein ID	Annotation	Sense Primer	Anti-sense Primer	Log2 Fold Change (E40/WT)	
					iTRAQ	qRT-PCR
Phenylpropanoid biosynthesis	Q5JMS4	Peroxidase	GCCAACACCACCGTCAAC	TGGAAGAACGCCGACTGG	1.16	0.89
	Q7XSV2	Peroxidase	CTCATCCAGGCGTTCAAG	CTTCTTCACCAGCACAGG	0.91	0.74
	Q94DM2	Class III peroxidase 22	TTGTCGTTGGGCTACTAC	AACTTCTCGCTCTTCTCG	0.71	0.46
	Q9AS12	Class III peroxidase 16	TCTTCCTCTTCTTCGCCTTC	ACGCCGCTGTTGTTCTTG	0.97	0.88
Porphyrin and chlorophyll metabolism	Q5Z8V9	Delta-aminolevulinic acid dehydratase	ATTCCAGGAGACCACCATC	CATCACGAGACTTGTAGACC	−0.62	−0.33
	Q6Z2T6	Geranylgeranyl diphosphate reductase	AGGAAGGTGAGGAAGATG	CAGGAAGAGACCATTGAC	−0.75	−0.42
Carbon fixation in photosynthetic organisms	P0C512	Ribulose bisphosphate carboxylase large chain	GGCAGCATTCCGAGTAAC	AAGTCCATCAGTCCAAACAG	−1.53	−1.57
	Q9SNK3	Glyceraldehyde-3-phosphate dehydrogenase	GCGAAGAAGGTCATCATCAC	GAGCGAGGCAGTTGGTTG	−0.84	−0.75

## Supplementary Materials

Supplementary materials can be found at https://www.mdpi.com/1422-0067/20/6/1294/s1.
